# Combined oral contraceptive utilization and uterine fibroid incidence: A prospective study in a cohort of African-American women

**DOI:** 10.1371/journal.pone.0303823

**Published:** 2024-05-23

**Authors:** Sarah R. Hoffman, Jennifer S. Smith, Michele Jonsson Funk, Michael G. Hudgens, Charles Poole, Wanda K. Nicholson, Donna D. Baird, Quaker E. Harmon

**Affiliations:** 1 Department of Epidemiology, University of North Carolina Gillings School of Global Public Health, Chapel Hill, North Carolina, United States of America; 2 Department of Biostatistics, University of North Carolina Gillings School of Global Public Health, Chapel Hill, North Carolina, United States of America; 3 Department of Obstetrics and Gynecology, University of North Carolina School of Medicine, Chapel Hill, North Carolina, United States of America; 4 University of North Carolina Center for Women’s Health Research, Chapel Hill, North Carolina, United States of America; 5 Program on Women’s Endocrine and Reproductive Health, University of North Carolina School of Medicine, Chapel Hill, North Carolina, United States of America; 6 Center for Health Promotion and Disease Prevention, Chapel Hill, North Carolina, United States of America; 7 Epidemiology Branch, National Institute of Environmental Health Sciences (NIEHS), Durham, Chapel Hill, North Carolina, United States of America; Future University, EGYPT

## Abstract

Published associations between combined oral contraceptive use and uterine fibroid development have lacked prospective imaging with ultrasound to distinguish between incident and prevalent fibroids. The Study of Environment, Lifestyle, and Fibroids prospectively followed fibroid-free, African-American women (the group with the highest disease burden in the U.S.) to identify incident cases. We examined associations between combined oral contraceptive use and the 40-month cumulative risk of fibroids. History of hormonal contraceptive use was collected via telephone interview at enrollment. Fibroid identification was performed using transvaginal ultrasonography at enrollment, and at 20 and 40-months of follow-up. Inverse probability weights for exposures and censoring were used to construct weighted risk ratios (wRR) and weighted risk different (wRD) estimators which control for differences in fibroid risk factors between exposure groups. In addition, unweighted fully adjusted log-binomial regression models (aRR) were run for comparison. Of the 1,308 participants in the analysis sample, 70% had used combined oral contraceptives and 17% developed fibroids by 40 months. We observed an inverse association between ever use of combined oral contraceptives and cumulative fibroid incidence (wRR: 0.78; 95% Confidence Interval (CI): 0.60, 1.00; wRD: -0.05, 95% CI: -0.11, 0; aRR: 0.76, 95% CI: 0.60, 0.98). Fibroid incidence was greater in participants who started using combined oral contraceptives after age 17 years than among younger initiators, though the restriction to ever-users made this estimate less precise (wRR: 1.25; 95% CI: 0.89, 1.76; wRD: 0.04, 95% CI: -0.02, 0.10). No consistent patterns of fibroid incidence were seen among ever-users for duration of, or years since, last combined oral contraceptives use.

## Introduction

More than 1 billion U.S. dollars are spent each year to treat uterine leiomyoma (fibroids) (non-malignant smooth muscle uterine tumors) [1]. Fibroid symptoms include bulk symptoms (pelvic pressure, urinary symptoms), pain, and heavy menstrual bleeding which can diminish quality of life and impact work productivity [2]. Fibroids can contribute to infertility and may be associated with preterm delivery [2,3]. Fibroids are the leading indication for hysterectomy [4]. Estimates based on ultrasound screening, irrespective of prior diagnosis or symptoms, suggest that more than 70% of women develop fibroids by menopause [5]. Though the prevalence of symptomatic fibroids is unknown, most women who are diagnosed have had symptoms for years [4]. Estimated annual costs of fibroids vary by assumptions about the prevalence of symptomatic fibroids, ranging from $5.89 to $34 billion [6]. The estimates include one aspect of quality of life, loss of productivity which was estimated to range from $5 –$17.2 billion. There are important racial disparities in the health burden associated with fibroids. In the U.S., African Americans develop fibroids an estimated 10 years earlier than White women, have a higher estimated incidence by the age of menopause, have larger fibroids at the time of diagnosis, and are 2–3 times more likely to require major medical or surgical procedures to treat fibroids [5,7–9].

While there has been advancement in surgical and medical treatment of symptomatic fibroids, considerably less research has focused on fibroid prevention. This is surprising given the high prevalence and public health burden of the disease. It is currently thought that estrogen and progesterone have complex, interrelated roles in fibroid tumor development and growth [10,11]. Progesterone is an important driver [12] of fibroid growth, and estrogen increases availability of progesterone receptors [11]. Injectable progestin (synthetic progesterone) contraceptives may offer lasting protection against uterine fibroids [13], and other forms of hormonal contraceptives are commonly used as a first-line treatment for fibroid symptoms [14,15].

Oral contraceptives (OCs) are generally composed of both estrogen and progestin [16]. Exposure to OCs is widespread in the U.S. [17–19]. Among sexually experienced, African-American women aged 15–44, 80% have used OCs in their lifetime [17]. Despite the availability of longer acting methods in the U.S., OCs remain the most commonly used form of hormonal contraception [20].

To date, published literature on the association between OC use and uterine fibroid development [21–32] have yielded mixed findings. Existing studies identified prevalent fibroid cases with cross-sectional ultrasound data, or relied on self-reported diagnoses of fibroid status which may be influenced by symptom severity and access to health care [33]. In addition, self-reported new diagnoses reflect detection of fibroids that likely developed years earlier. Thus, the current literature is largely unable to establish temporality in the reported relationships between OCs and fibroids. Further, existing studies have only compared any history of oral contraceptive use to never use. Comparing the incidence of fibroids associated with different durations of use and other utilization characteristics would be valuable since most women use oral contraceptives in their lifetime [17–19]. Finally, few studies [21,25–27] included ≥10% African-American participants, the population with the largest fibroid burden both in terms of occurrence and severity [34–37].

We sought to address these limitations by examining the longstanding question of how OC use influences uterine fibroid development, using data from the Study of Environment, Lifestyle, and Fibroids (SELF) [8]. SELF is the first large prospective, ultrasound-based study of risk factors for uterine fibroid incidence, and exclusively enrolled young, African-American participants [8]. Specifically, we examined the associations between different characteristics of oral contraceptive use and incident fibroids at ~40 months of follow-up among participants who were fibroid-free at enrollment.

## Materials and methods

This study was completed as part of a PhD dissertation at the University of North Carolina at Chapel Hill [38], which is publicly available at the Carolina Digital Repository (https://cdr.lib.unc.edu/).

### Study population

The Study of Environment, Lifestyle & Fibroids (SELF) is a prospective cohort study of 1,693 young (23–35 years), African-American women living in the Detroit, Michigan area. SELF was designed to investigate risk factors for uterine fibroid incidence and growth [8,39–41]. Recruitment and baseline data collection were completed in 2010–2012 [8]. Participants were recruited from the Detroit area via local media commercials and advertisements, brochures at healthcare clinics, information booths at community events, and via Henry Ford Health (HFH) [8]. The primary eligibility requirements were age (23–35 years), self-identified African American/Black, and having no prior clinical diagnosis of uterine fibroids. Additional eligibility criteria included U.S. residence, a willingness to return for clinic visits over 5 years and provide contact information. Exclusion criteria included a prior diagnosis of fibroids, receipt of radiation or chemotherapy for treatment of cancer, and prior diagnosis of autoimmune disorders requiring medication [8]. Participants had ultrasounds and questionnaire data collected at enrollment and at each follow-up, scheduled at approximately 20-month intervals. This analysis includes data collected through the 40-month follow-up. Study retention rate was high at >85% for the 40-month follow-up.

All participants received a study orientation in-person or over the phone describing all aspects of the study including a detailed walk-through of the consent form with an opportunity to ask questions. Following this orientation all participants provided written informed consent witnessed by trained study staff. SELF was approved by the institutional review boards of the National Institute of Environmental Health Sciences and Henry Ford Health.

### Data collection

All exposure and covariate data were self-reported using telephone or computer-assisted questionnaires, with the exception of BMI, which was calculated using height and weight as measured at the enrollment clinic visit.

#### Hormonal contraceptive use

History of hormonal contraceptive (HC) use was collected via telephone interview as part of an enrollment questionnaire. Participants were asked if they had ever used each of the following types of HC: “birth control pills” (OCs), “mini-pill” (progestin-only OCs), hormonal implant, hormonal patch, vaginal ring, “hormone shots like Depo-Provera^®^”, and hormonal intrauterine devices (H-IUD). Brief descriptions and examples of common brand names were provided for hormonal implants and shots.

For each HC type (and separately for each H-IUD), participants were asked about their age at first use, whether or not they were currently using and how old they were when they stopped using. For age at first use, participants were asked, “How old were you when you started using birth control pills, whether or not it was to prevent pregnancy?” Participants who had used OCs were asked whether or not they had ever stopped using for a month or longer, between initial start and last stop (or study enrollment if currently using), and if so, how much of that time was spent on OCs (“very little of that time,” “less than half of that time,” “about half,” “more than half,” “most of that time”). Participants who used the “mini-pill,” implant, patch, ring, or shot were asked to state the total number of months and years that they had used each method prior to study enrollment.

#### Uterine fibroids

Fibroid identification was performed using transvaginal ultrasonography–the current standard of care for uterine fibroid assessment [42,43]. Transvaginal ultrasonography has 99% sensitivity and 91% specificity for detecting uterine fibroids [44]. If any fibroids with at least one diameter of 0.5 cm or greater were detected, the largest six fibroids were measured in three separate passes through the uterus. Ultrasound examinations were conducted by a consistent group of registered diagnostic sonographers with at least 3 years of experience in gynecologic sonography. The study sonographers were given initial and refresher trainings including care in distinguishing fibroids from other pathologic changes in the uterus including adenomyosis and polyps, protocol for conducting the exam, and recording the data. Video and still images were archived and an 8% sample for each sonographer, oversampled for fibroid cases, was reviewed every month by the lead sonographer [8].

Among those fibroid-free at the enrollment ultrasound, incident fibroid by the 40-month follow-up was “yes” if fibroids were seen at either the 20- or 40-month follow-up. It was “no” if no fibroids were seen at the 20- or 40-month follow-up. Participants with no follow-up ultrasounds as well as those with no fibroids at 20-months but no 40-month ultrasound were excluded during analysis because their cumulative incidence at 40 months could not be assessed. Of those fibroid-free at enrollment (n = 1,308), 198 were missing the outcome variable. Reasons for missing data for the outcome included missing the study visit (n = 190), non-fibroid related hysterectomy (n = 7), and poor ultrasound quality (n = 1). Excluded individuals were included in our propensity score calculations, and inverse probability of censoring weights were applied in order to upweight similar individuals with complete data in the calculations of our estimates (see S1 Appendix in S1 File).

#### Exposure classification

Ever users of combined oral contraceptives (COCs, containing both estrogen and progestin) were participants who answered “yes” to any use of oral contraceptives, excluding those who used only the mini-pill [45]. Participants who reported using “the pill” were considered to have used COCs if they answered “No” or “I don’t know” to “Have you ever used a progesterone-only birth control pill, or “mini-pill”, such as Micronor, Nora-BE, or Ovrette?” Age at first COC use was dichotomized into <17 and ≥17 based on findings in previous literature [21,29].

Duration of use estimates were calculated in three steps: (1) Subtract age at first OC use from age at last OC use, or age at enrollment for current users, (2) Subtract years and months on the mini-pill, (3) Multiply by the self-reported proportion of time spent using OCs. Weights were applied as follows: 10% for “very little of that time,” 25% for “less than half of that time,” 50% for “about half,” 75% for “more than half,” 90% for “most of that time,” and 100% for those who had no interruption in their use. When age at first use and age at last use were identical, a duration of six months was assigned.

For former COC users, years since last COC use was calculated by subtracting self-reported age at last pill use from age at enrollment. Current users were assigned a “years since last use” value of 0. For n = 64 participants who used both combined and progestin-only OCs, years since last COC use specifically could be determined for 31 subjects. Years since last use of COCs could not be determined for the remaining 33 participants due to limitations in the enrollment questionnaire.

Joint categories of duration of use and years since last use were created. Duration of use was characterized as short (<2 years) or long (≥2 years) and years since last use was characterized as recent (<5 years) or past (≥5 years), based on available data, creating four joint categories.

#### Covariates

The covariate set for all weight calculations included the following variables categorized as follows: age in years (continuous), age at menarche (≤10 years, yes/no), Depo-Provera use (4 categories of duration and years since last use), implant and H-IUD use (use ≥ 24 months, yes/no), years since last birth (0–4, 5–9, 10+ years or no birth), parity (nulliparous or never pregnant, 1 birth, 2 births, ≥ 3 births), BMI (in four categories: >30, and remaining values according to tertile), and education (Bachelor’s degree or higher, yes/no). In addition to these covariates, censoring weights also included the exposure of interest, annual household income (<$20,000, $20-$50,000, ≥$50,000), baseline employment status (not employed, employed <30 hrs/wk, employed ≥30 hrs/wk), smoking history (never, former, current <10/day, current ≥10/day), and history of heavy “gushing” type menstrual bleeding (yes/no).

#### Statistical analyses

All data management and analyses were performed in SAS 9.4 (SAS Institute, Cary, NC, USA). We used inverse probability weights methods to account for differences in fibroid risk factors and possible selection bias between exposure groups, and we report this propensity score-based methods in accordance with the recommendations provided by Ali et al. (2014) [46] in the Journal of Clinical Epidemiology in Supplemental Appendices 1–3. In brief, inverse probability weights were constructed for all COC exposures, and for censoring. Standardized morbidity ratio weights were constructed for ever-COC use. Similarly, we used weights as described in S1 Appendix in S1 File to estimate associations among all COC users. Absolute standardized differences were used to assess covariate balance with the conventional a priori threshold of 0.1. Weighted log-binomial regression models were used to estimate risk ratios and risk differences for uterine fibroids. Confidence intervals for weighted models were generated using robust variance (“sandwich”) estimator by use of the SAS REPEAT statement. Unweighted multivariable log-binomial regression models, with the same set of covariates used in the weighted models, were run for comparison. As recommended by the American Statistical Association leadership, associations were interpreted based on the strength of the estimate, width of the confidence interval (precision) and consistency across models, not on statistical significance at p≤0.05 [47].

We first compared ever to never users of COCs, and then compared different levels of COC use (e.g., age at first use, duration of use, etc.) among the COC users. This is in contrast to existing studies, which compared different levels of COC use to never-use–a comparison that is unlikely to be useful given that distinctions by duration of use and time since last use are relevant primarily to ever COC users.

## Results

Among the 1,308 SELF participants who were fibroid-free at enrollment, median age at enrollment was 28 years [IQR: 25, 31] (Table 1). Very few baseline characteristics differed between ever and never users with the exception that ever users of COCs more frequently reported higher annual household income, higher educational attainment, never smoking, heavy menstrual bleeding, and childbirth, and less frequently reported Long/Recent (>24 months, within 8 years) use of Depo-Provera (Table 1).

**Table 1:** Characteristics of 1,308 fibroid-free participants enrolled in SELF in 2010–2012 (Detroit, MI, USA), stratified by ever use of combined oral contraceptives (COCs) at enrollment.

	Never used COCs	Ever used COCs	Total
	N = 395	N = 913	N = 1,308
	n (%)	n (%)	n (%)
**Age at enrollment (years)**			
Median [IQR]	28 [25, 30]	29 [26, 32]	28 [25, 31]
**Annual household income**			
< $20,000	225 (57)	382 (42)	607 (46)
$20,000 to $50,000	119 (30)	370 (41)	489 (37)
≥$50,000	48 (12)	153 (17)	201 (15)
Don’t know/refused	3 (<1)	8 (<1)	11 (<1)
**Baseline employment status**			
Not employed	190 (48)	328 (36)	518 (40)
Employed, < 30 hrs/wk	55 (14)	117 (13)	172 (13)
Employed, ≥30 or more hrs/wk	150 (38)	465 (51)	615 (47)
Don’t know/on leave/no usual hours	0 (0)	3 (<1)	3 (<1)
**Education** *			
Bachelors/master’s/PhD	80 (20)	256 (28)	336 (26)
**Body mass index (kg/m** ^**2**^ **)**			
< 25	90 (23)	170 (19)	260 (20)
25–29	80 (20)	199 (22)	279 (21)
≥30	225 (57)	544 (60)	769 (59)
**Smoking history**			
Never smoked	268 (68)	691 (76)	959 (73)
Former smoker	26 (7)	71 (8)	97 (7)
Current smoker (< 10/day)	75 (19)	118 (13)	193 (15)
Current smoker (≥ 10/day)	26 (7)	33 (4)	59 (5)
**Age at menarche**			
≤10 years	65 (16)	161 (18)	226 (17)
**Heavy menstrual bleeding**			
Ever had heavy gushing type bleeding	128 (32)	338 (37)	466 (36)
**Reproductive history**			
Nulliparous or never pregnant	162 (41)	315 (35)	477 (36)
1 birth	95 (24)	249 (27)	344 (26)
2 births	63 (16)	183 (20)	246 (19)
≥ 3 births	75 (19)	166 (18)	241 (18)
**Years since last birth**			
0–4 years	142 (36)	325 (36)	467 (36)
5–9 years	63 (16)	202 (22)	265 (20)
Nulliparous or ≥ 10 years ago	190 (48)	386 (42)	576 (44)
**Depo-Provera history**			
Never used Depo-Provera	220 (56)	495 (54)	715 (55)
Short/Past (≤ 24 months, > 8 years ago)	18 (5)	95 (10)	113 (9)
Long/Past (> 24 months, > 8 years ago)	15 (4)	32 (4)	47 (4)
Short/Recent (≤ 24 months, within 8 years)	59 (15)	155 (17)	214 (16)
Long/Recent (> 24 months, within 8 years)	83 (21)	136 (15)	219 (17)
**Implant and H-IUD history**			
Used ≥ 24 months	25 (6)	68 (7)	93 (7)

*Education was missing for n = 1 participants in the never used COCs group.

**Abbreviations:** SELF, Study of Environment, Lifestyle, and Fibroids; COC, combined oral contraceptives; IQR, interquartile range; hrs, hours; wk, week; PhD, Doctor of Philosophy; kg/m^2^, kilograms per square meter; H-IUD, hormonal intrauterine device.

Among the 913 (70%) SELF participants who had ever used COCs, 320 (35%) reported initiating COCs prior to age 17 years, and most (73%) used COCs for less than 5 years in total (Table 2). Relatively few (24%) were currently using COCs at study enrollment. Nearly half (48%) had last used COCs 5 or more years ago (Table 2). Unweighted and weighted risks for developing fibroids during the follow-up period are provided in the “Incident Fibroid Cases” columns of Table 2. Approximately 17% (n = 221) developed fibroids by the 40-month follow-up.

**Table 2:** Combined oral contraceptive utilization and fibroid incidence in 1,308 fibroid-free women who enrolled in SELF in 2010–2012 (Detroit, MI, USA).

Incident Fibroid Cases		Risk Ratios (95% CI)			Risk Differences (95% CI)	Incident Fibroid Cases
COC Use	n (%)	Age-Adjusted	Fully Adjusted*		Fully Adjusted*	n (%)*
	Unweighted	MVR	IPW/SMR	MVR	IPW/SMR	IPW/SMR
**Among all participants** (N = 1,308)						
Never (N = 395)	77 (19)	Ref.	Ref.	Ref.	Ref.	178 (23)^†^
Ever (N = 913)	144 (16)	0.77 (0.60, 0.99)	0.78 (0.60, 1.00)^†^	0.76 (0.60, 0.98)	-0.05 (-0.11, 0)^†^	140 (18)^†^
**Among ever-users** (N = 913)						
Age at first use (years)						
< 17 (N = 320)	42 (13)	Ref.	Ref.	Ref.	Ref.	116 (15)
≥ 17 (N = 593)	102 (17)	1.25 (0.90, 1.74)	1.25 (0.89, 1.76)	1.20 (0.86, 1.67)	0.04 (-0.02, 0.10)	150 (19)
Duration of use (years)						
< 1 (N = 278)	34 (12)	Ref.	Ref.	Ref.	Ref.	117 (15)
1–1.99 (N = 164)	29 (18)	1.49 (0.95, 2.33)	1.49 (0.94, 2.38)	1.43 (0.92, 2.22)	0.07 (-0.01, 0.16)	168 (23)
2–4.99 (N = 229)	34 (15)	1.18 (0.76, 1.82)	1.10 (0.69, 1.74)	1.11 (0.72, 1.70)	0.01 (-0.06, 0.09)	126 (17)
≥ 5 (N = 242)	47 (19)	1.48 (0.99, 2.21)	1.30 (0.83, 2.04)	1.23 (0.81, 1.86)	0.05 (-0.03, 0.12)	157 (20)
Years since last use^‡^						
Current user (N = 216)	36 (17)	Ref.	Ref.	Ref.	Ref.	155 (20)
1–2 (N = 134)	25 (19)	1.18 (0.75, 1.85)	1.23 (0.72, 2.10)	1.23 (0.79, 1.92)	0.05 (-0.07, 0.17)	174 (25)
3–4 (N = 95)	16 (17)	1.01 (0.60, 1.70)	0.96 (0.50, 1.85)	1.09 (0.64, 1.84)	-0.01 (-0.14, 0.12)	154 (19)
≥ 5 (N = 441)	64 (15)	0.77 (0.53, 1.12)	0.87 (0.55, 1.37)	0.91 (0.62, 1.33)	-0.03 (-0.12, 0.06)	129 (17)
Characteristics of use^‡^^§^						
Short/past (N = 256)	37 (14)	Ref.	Ref.	Ref.	Ref.	136 (18)
Short/recent (N = 172)	25 (15)	1.25 (0.78, 2.00)	0.97 (0.58, 1.62)	1.09 (0.68, 1.74)	-0.01 (-0.1, 0.09)	123 (18)
Long/past (N = 185)	27 (15)	0.96 (0.61, 1.51)	0.92 (0.55, 1.54)	0.86 (0.55, 1.35)	-0.01 (-0.1, 0.08)	124 (17)
Long/recent (N = 273)	52 (19)	1.39 (0.95, 2.04)	1.19 (0.77, 1.83)	1.13 (0.76, 1.68)	0.03 (-0.05, 0.12)	166 (22)

*Weighted (IPW/SMR) or Adjusted (MVR) for age in years (continuous), age at menarche (<11 years), Depo-Provera duration of and years since last use (never use, short/past, long/past, short/recent, long/recent), total implant and H-IUD duration of use (≥24 months), years since last birth (<5 years, 5–9.99 years, ≥10 years and no birth), parity (nulliparous or never pregnant, 1 birth, 2 births, ≥3 births), BMI (≥30, remaining values according to tertile), education (Bachelor’s degree or higher). Weighted risk ratios, risk differences, and incident fibroid case counts are shown in the columns corresponding to this footnote.

A total of n = 198 women were censored, including n = 136 COC users. Inverse probability of censoring weights were applied to upweight individuals most likely to have been censored who remained in the study. The model for probability of censoring included the exposure of interest (e.g., duration of use), all covariates used in the IPW/SMR models, with the addition of annual household income, baseline employment status, smoking history, and history of heavy gushing type bleeding.

^†^The weighted estimate for Ever-use was weighted according to the covariate distribution of COC users; i.e., standardized morbidity ratio [SMR] weighting was employed for this exposure.

^‡^Excludes n = 27 participants who used both pill and mini-pill, for whom years since last COC use could not be distinguished.

^§^Duration of use was characterized as short (< 2 years) or long (≥ 2 years). Years since last use was characterized as recent (< 5 years) or past (≥ 5 years).

**Abbreviations:** SELF, Study of Environment, Lifestyle, and Fibroids; CI, confidence interval; MVR, multivariable logistic regression; IPW, inverse probability weighting; SMR, standardized morbidity ratio; H-IUD, hormonal intrauterine device; BMI, body mass index.

**Fig 1:**
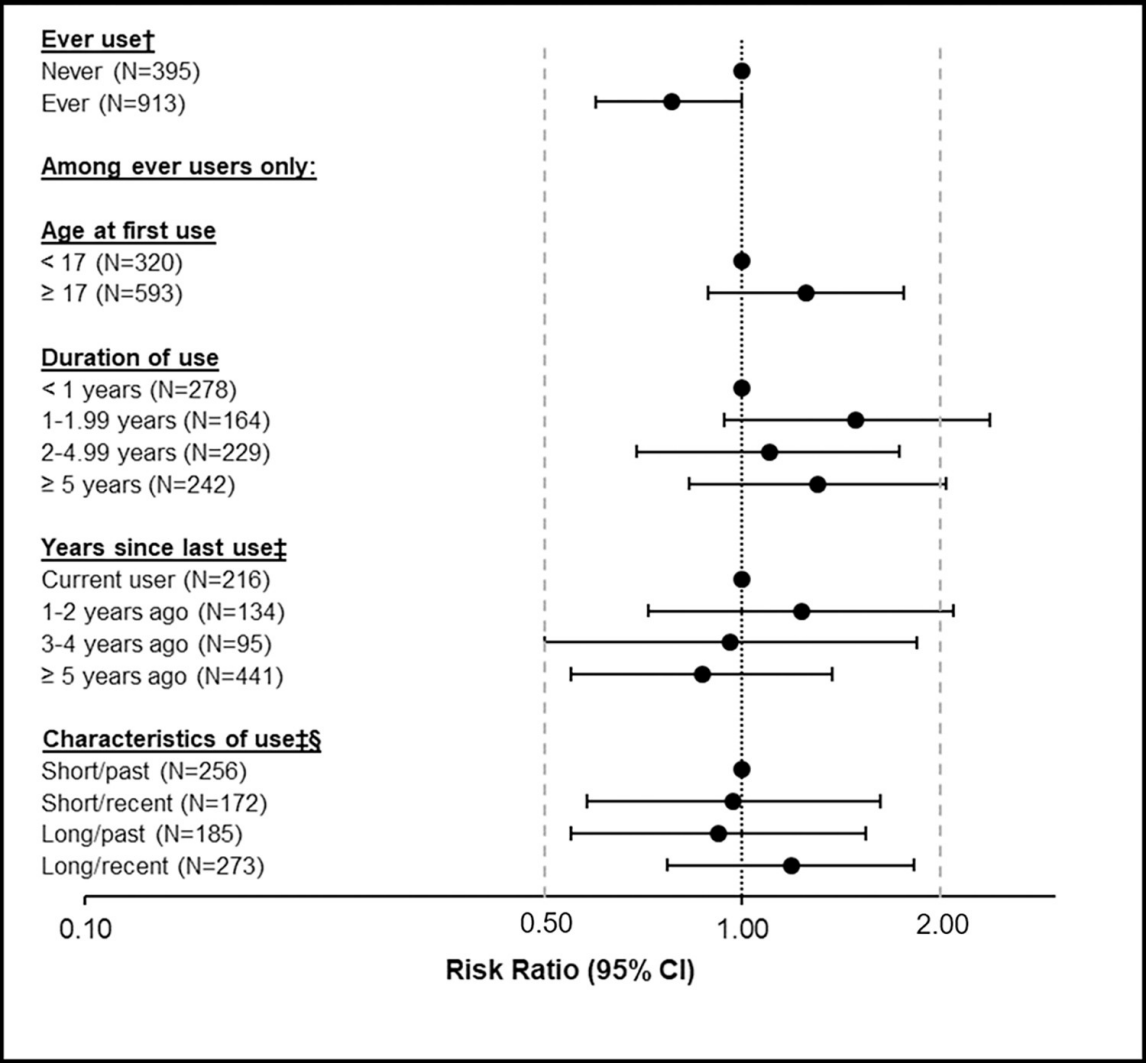
IPW/SMR* associations between levels of combined oral contraceptive utilization and fibroid incidence. *Weighted (IPW/SMR) for age in years (continuous), age at menarche (<11 years), Depo-Provera duration of and years since last use (never use, short/past, long/past, short/recent, long/recent), total implant and H-IUD duration of use (≥24 months), years since last birth (<5 years, 5–9.9 years, ≥10 years and no birth), parity (nulliparous or never pregnant, 1 birth, 2 births, ≥3 births), BMI (≥30, remaining values according to tertile), education (Bachelor’s degree or higher). A total of n = 198 women were censored, including n = 136 COC users. Inverse probability of censoring weights were applied to upweight individuals most likely to have been censored who remained in the study. The model for probability of censoring included the exposure of interest (e.g., duration of use), all covariates used in the IPW/SMR models, with the addition of annual household income, baseline employment status, smoking history, and history of heavy gushing type bleeding. ^†^The weighted estimate for Ever-use was weighted according to the covariate distribution of COC users; i.e., standardized morbidity ratio [SMR] weighting was employed for this exposure. ^‡^Excludes n = 27 participants who used both pill and mini-pill, for whom years since last COC use could not be distinguished. ^§^Duration of use was characterized as short (< 2 years) or long (≥ 2 years). Years since last use was characterized as recent (< 5 years) or past (≥ 5 years). **Abbreviations:** IPW, inverse probability weighting; SMR, standardized morbidity ratio; SELF, Study of Environment, Lifestyle, and Fibroids; CI, confidence interval; MVR, multivariable logistic regression; H-IUD, hormonal intrauterine device; BMI, body mass index.

At the 40-months’ follow-up, we observed a possible inverse association between ever use of COCs and fibroid cumulative incidence (wRR 0.78, 95% CI: 0.60, 1.00; wRD -0.05, 95% CI: -0.11, 0; Table 2, Fig 1, S3 and S4 Appendices in S1 File). Among ever users of COCs, age at first use ≥17 years was associated with an elevated risk of fibroid incidence (wRR: 1.25; 95% CI: 0.89, 1.76; wRD: 0.04, 95% CI: -0.02, 0.10) compared to those initiating COCs prior to age 17, although the estimate was imprecise. Comparing each level of duration of COC use to <1 year of use, weighted risk ratios varied from 1.10 to 1.49, with elevated risk in every category other than the shortest-term use (Table 2, Fig 1, S3 and S4 Appendices in S1 File). Weighted risk ratios comparing each level of years since last use to current use ranged from 0.87 to 1.23, declining with increased years since last use (Table 2, Fig 1, S3 and S4 Appendices in S1 File). Compared to short-term use in the past (<2 years of COC use, ≥5 years age), other cross-classified patterns of use demonstrated no clear trend.

## Discussion

In this study of 1,308 young, African-American participants living in Detroit, who were fibroid-free at enrollment, we found an inverse relationship between ever use of COCs and incidence of uterine fibroids. Among COC users, the estimate for age at first use ≥17 years was suggestive of a positive association with fibroid incidence, while the estimates for duration of use and years since last use were imprecise and inconsistent across exposure categories. Four prior case-control studies reported odds ratios comparing ever to never oral contraceptive users [23,28,31,32]. Two studies [28,32] reported no association, one study [23] reported a positive association of OR = 1.4 (95% CI: 0.9–2.1; adjusted for age but no other confounders), and one study [31] reported an inverse association of OR = 0.76 (95% CI: 0.66–0.92). The inverse association with prevalent fibroids is similar to our incidence wRR of 0.78 (95% CI: 0.60, 1.00). All four prior studies defined cases as surgically treated leiomyomas, used other hospitalized patients as controls, and were conducted ≥20 years ago in Italy [23,28,32] or Thailand [31], when oral contraceptive formulations and the availability of other forms of HC were likely different from those available to the participants in SELF. The cohort of participants in SELF have had access, starting in their early teens, to 2^nd^ and 3^rd^ generation oral contraceptives which include lower doses of ethinyl estradiol and newer progestins at lower doses. Further, these prior studies identified prevalent cases of fibroids rather than newly developed fibroids among fibroid-free participants. Thus, in these studies the timing of HC use could have been after fibroids had developed.

While two prior prospective studies that identified cases at time of their first clinical diagnosis reported an increased risk of fibroid incidence associated with COC initiation before age 17 years [21,29], we did not observe this association in our data. While these studies compared each age at first use category to never use, our age at first use comparisons were restricted to ever COC users.

Though our study design and population differed considerably, our imprecise findings for duration of use and years since last use of COCs are in concurrence with most prior literature [21,23,24,26,29,30,32], including the Black Women’s Health Study (BWHS) [21]. However, three prior studies did report an inverse association for longer duration of use of OCs [22,24,28], and one study reported that longer years since last OC use (>5 year versus ≤5 years) was associated with increased uterine fibroid prevalence (unadjusted p < 0.01) [22]. Never users served as the referent for two of these three studies [24,28], and the third study only reported crude results without a clear referent group [22].

Prior laboratory work confirms that fibroids are hormonally dependent tumors which require progesterone [48], while estrogen is important for maintaining progesterone receptors [49]. Thus, there is biologic plausibility for COCs influencing fibroid development. Also, fibroid development may be influenced by inflammatory pathways [50], and concentrations of C-reactive protein (a marker of inflammation) increase with increasing BMI [50]. This raises questions about a possible BMI interaction in an association between COCs and fibroid incidence. However, this epidemiologic study did not have accurate data on a participant’s BMIs prior to enrollment when the majority of COC exposure occurred, nor did we collect fibroid/myometrial tissue or other biological measurements to evaluate mechanistic hypotheses.

### Strengths and limitations

Ours is the first large prospective, ultrasound-based study to examine the association between COC use and uterine fibroid incidence in young, African-American participants. SELF performed ultrasounds in study participants at enrollment and follow-up, regardless of symptoms or health care access. Analyses were restricted to participants without fibroids at enrollment, allowing for more confidence that the COC exposure preceded the outcome, an important condition for causality [51]. Prior studies used multivariable regression models and most often compared different levels of COC use (e.g., 10 years of use) to never users. Comparison with never users may not be the most relevant comparison as women are unlikely to choose between a specific level of COC use (e.g., duration of use 3–4 years) and never using COCs at all. Analogous to the counterfactual conundrum of smoking [52,53], we are unlikely to ever live in a world in which no women ever use COCs. Therefore, our comparisons of different levels of use within-COC users are likely to be more relevant to prescribers, patients, and policymakers [52–54].

Our findings should be considered in light of our study’s limitations. First, all exposure and covariate data (with the exception of BMI) were self-reported which could have resulted in inaccuracies for sensitive covariates such as household income and covariates that may be difficult to recall (e.g., duration of use for individual hormonal contraceptives). Similarly, we did not have exact duration of use estimates for COCs and had to use limited available information to estimate duration of use. However, self-reported history of OC use, as collected by telephone interview, has been found to be reliable when compared to automated pharmacy dispensing data [55]. The specific COCs that were used before baseline were not queried, so we were not able to examine associations between specific formulations and uterine fibroids. Second, while uterine fibroids were captured prospectively, exposure information was limited to baseline, raising the possibility of prevalent-user bias [56]. Further, we did not examine duration of use, time-since-last use, or age at first use as continuous variables, but instead used clinically- and operationally pragmatic cut points allowing for analysis with the available sample size. In the case of age at first use, a cut point was chosen to be consistent with prior literature [21,25,29]. Additionally, we conducted an ever-never comparison for consistency with prior literature, though the utility of this comparison is questionable considering that most women use OCs in their lifetimes [17–19]. Our never-users comparison group includes participants who were not contracepting or have chosen other methods of contraception and who may differ from the ever users in ways that are unmeasured and not accounted for in the weighted analysis. Additionally, the follow-up period for this study was limited to 40 months; this analysis did not examine longer-term effects of COC use on fibroid incidence. Finally, this study focuses on a specific ethnicity in a specific geographic area and timeframe, and findings may not be generalizable to other populations with different distributions of hormonal contraceptive formulations or utilization patterns. We encourage continued research into the association between COC use and fibroids in different populations as new COC formulations become available.

Despite these limitations, this is the first study to examine truly incident fibroids in participants who all came of age after the transition from COC formulations with high hormonal doses to low dose formulations. Specifically, we overcame the misclassification of those with undiagnosed fibroids who are included in “non-cases” when clinical populations, or self-reported fibroid status are used to differentiate cases and controls. Also, given that clinical diagnosis (outcome in prior prospective studies) often occurs years after the development of fibroids, some women will use OCs to manage fibroid symptoms even if they don’t know that they have fibroids. This can create strong bias in exposure outcome associations.

The inverse association seen for ever use of COCs and fibroid incidence should be reassuring to care providers and those seeking contraceptive options, even at a young age. Previous studies which suggest an increased risk of prevalent fibroids with use of COCs [23], and increased risk of incident, clinically diagnosed, fibroids with use of COCs before the age of 17 years [21,29], were completed among women who likely used COCs with a higher dose of estrogen and older, higher doses of progestins. Current COC formulations do not appear to carry a similar risk profile. Finally, replication studies are needed; if the inverse association of ever use is replicated, more detailed study of life-course patterns of use are necessary to understand the driver(s) of the inverse association.

## Conclusion

Though ever use of COCs showed an inverse association with uterine fibroid development, estimates for the COC-use factors that might therefore have been expected to be associated with reduced fibroid risk were less precise and directionally inconsistent (e.g., wRR >1 for longer durations of use and more recent use; Table 2, Fig 1, S3 and S4 Appendices in S1 File). Contrary to prior reports [21,34] which relied on never users as the referent group and showed increased self-reported fibroid incidence among young (age <17 years) COC initiators, we observed a higher incidence of fibroids in COC users who initiated COC use on or after age 17 years, although the estimate was imprecise. Estimates for duration of use or years since last COC use were limited in precision due to study size. Further studies should be conducted in larger data sets that would lead to estimates that are more precise and that build upon the methodological improvements represented by this study. Additional tissue-based studies using the new formulations of COCs are also needed to clarify the biological mechanisms at play, particularly among early COC initiators.

## Supporting information

S1 Checklist STROBE statement—checklist of items that should be included in reports of observational studies. (DOCX)

S1 File (DOCX)

## Abbreviations

aRR: adjusted risk ratio
COC: combined oral contraceptive(s); combined oral contraception
HC: hormonal contraceptive(s); hormonal contraception
H-IUD: hormonal intrauterine device(s)
IQR: interquartile range
OC: oral contraceptive(s); oral contraception
SELF: study of environment, lifestyle, and fibroids
wRD: weighted risk difference
wRR: weighted risk ratio

## References

[1] Gliklich R, Leavy M, Velentgas P, Campion D. Identification of future research needs in the comparative management of uterine fibroid disease: A report on the priority-setting process, preliminary data analysis, and research plan. [Internet]. AHRQ Effective health care program research reports. 2011. Available from: https://scholar.google.com/scholar?q=gliklich+identification+of+future+research+fibroids&btnG=&hl=en&as_sdt=0%2C26#1.

[2] Donnez J, Dolmans M-M. Uterine fibroid management: from the present to the future. Hum Reprod Update [Internet]. 2016 Nov [cited 2017 Feb 19];22(6):665–86. Available from: http://www.ncbi.nlm.nih.gov/pubmed/27466209. doi: 10.1093/humupd/dmw023 27466209 PMC5853598

[3] Lee HJ, Norwitz ER, Shaw J. Contemporary management of fibroids in pregnancy. Rev Obstet Gynecol [Internet]. 2010 [cited 2017 Feb 19];3(1):20–7. Available from: http://www.ncbi.nlm.nih.gov/pubmed/20508779. 20508779 PMC2876319

[4] Stewart EA, Laughlin-Tommaso SK, Catherino WH, Lalitkumar S, Gupta D, Vollenhoven B. Uterine fibroids. Nat Rev Dis Prim. 2016;2(June). 10.1038/nrdp.2016.43 27335259

[5] Baird D, Dunson DB, Hill MC, Cousins D, Schectman JM. High cumulative incidence of uterine leiomyoma in black and white women: Ultrasound evidence. Am J Obstet Gynecol [Internet]. 2003;188(1):100–7. Available from: http://linkinghub.elsevier.com/retrieve/pii/S0002937802714294. doi: 10.1067/mob.2003.99 12548202

[6] Cardozo ER, Clark AD, Banks NK, Henne MB, Stegmann BJ, Segars JH. The estimated annual cost of uterine leiomyomata in the United States. Am J Obstet Gynecol [Internet]. 2012 [cited 2023 Dec 13];206(3):211.e1-211.e9. Available from: https://pubmed.ncbi.nlm.nih.gov/22244472/. doi: 10.1016/j.ajog.2011.12.002 22244472 PMC3292655

[7] Laughlin-Tommaso SK, Jacoby VL, Myers ER. Disparities in Fibroid Incidence, Prognosis, and Management. Obstet Gynecol Clin North Am [Internet]. 2017 Mar [cited 2017 Jul 21];44(1):81–94. Available from: http://linkinghub.elsevier.com/retrieve/pii/S0889854516300961. doi: 10.1016/j.ogc.2016.11.007 28160895

[8] Baird DD, Harmon QE, Upson K, Moore KR, Barker-Cummings C, Baker S, et al. A Prospective, Ultrasound-Based Study to Evaluate Risk Factors for Uterine Fibroid Incidence and Growth: Methods and Results of Recruitment. J Womens Health (Larchmt). 2015;24(11):907–15. doi: 10.1089/jwh.2015.5277 26334691 PMC4649767

[9] Al-Hendy A, Myers ER, Stewart E. Uterine Fibroids: Burden and Unmet Medical Need. Semin Reprod Med [Internet]. 2017 Nov 1 [cited 2023 Dec 13];35(6):473–80. Available from: https://pubmed.ncbi.nlm.nih.gov/29100234/. doi: 10.1055/s-0037-1607264 29100234 PMC6193285

[10] Wise LA. Epidemiology of Uterine Fibroids: From Menarche to Menopause. Clin Obstet Gynecol. 2016. doi: 10.1097/GRF.0000000000000164 26744813 PMC4733579

[11] Reis FM, Bloise E, Ortiga-Carvalho TM. Hormones and pathogenesis of uterine fibroids. Best Pract Res Clin Obstet Gynaecol [Internet]. 2015;1–12. Available from: http://linkinghub.elsevier.com/retrieve/pii/S1521693415002291. doi: 10.1016/j.bpobgyn.2015.11.015 26725037

[12] Rothman KJ, Greenland S, Lash TL. Modern Epidemiology, 3rd Edition. Philadelphia, PA: Lippincott Williams & Wilkins; 2008.

[13] Harmon QE, Patchel SA, Zhao S, Umbach DM, Cooper TE, Baird DD. Depot Medroxyprogesterone Acetate Use and the Development and Progression of Uterine Leiomyoma. Obstet Gynecol [Internet]. 2022 May 1 [cited 2023 May 11];139(5):797–807. Available from: https://pubmed.ncbi.nlm.nih.gov/35576339/. doi: 10.1097/AOG.0000000000004745 35576339 PMC9015023

[14] Yao X, Stewart EA, Laughlin-Tommaso SK, Heien HC, Borah BJ. Medical therapies for heavy menstrual bleeding in women with uterine fibroids: a retrospective analysis of a large commercially insured population in the USA. BJOG An Int J Obstet Gynaecol. 2017;124(2):322–30. doi: 10.1111/1471-0528.14383 27770484 PMC5736004

[15] ACOG Committee on Practice Bulletins—Gynecology. Management of Symptomatic Uterine Leiomyomas: ACOG Practice Bulletin, Number 228. Obstet Gynecol [Internet]. 2021 Jun 1 [cited 2023 May 11];137(6):e100–15. Available from: https://pubmed.ncbi.nlm.nih.gov/34011888/. doi: 10.1097/AOG.0000000000004401 34011888

[16] Dhont M. History of oral contraception. Eur J Contracept Reprod Heal Care [Internet]. 2010 Dec 22 [cited 2017 Feb 12];15(sup2):S12–8. Available from: http://www.ncbi.nlm.nih.gov/pubmed/21091163. doi: 10.3109/13625187.2010.513071 21091163

[17] Daniels K, Mosher WD, Jones J. Contraceptive Methods Women Have Ever Used: United States, 1982–2010. Natl Health Stat Report [Internet]. 2013 [cited 2016 Sep 17];(62):1–15. Available from: http://www.cdc.gov/nchs/data/nhsr/nhsr062.pdf. 24988816

[18] Daniels K, Daugherty J, Jones J, Mosher W. Current contraceptive use and variation by selected characteristics among women aged 15–44: United States, 2011–2013. Natl Heal Stat Rep. 2015;(86):1–14. 26556545

[19] Jones RK. Beyond Birth Control: The Overlooked Benefits Of Oral Contraceptive Pills [Internet]. 2011 [cited 2016 Sep 17]. Available from: https://www.guttmacher.org/report/beyond-birth-control-overlooked-benefits-oral-contraceptive-pills.

[20] Jones J, Mosher W, Daniels K. Current contraceptive use in the United States, 2006–2010, and changes in patterns of use since 1995. Natl Health Stat Report. 2012;1980(60):1–26. 24988814

[21] Wise LA, Palmer JJR, Harlow BLB, Spiegelman D, Stewart EEA, Adams-Campbell LLL, et al. Reproductive Factors, Hormonal Contraception, and Risk of Uterine Leiomyomata in African-American Women: A Prospective Study. Am J Epidemiol. 2004;159(2):113–23. doi: 10.1093/aje/kwh016 14718211 PMC1847588

[22] Marino JL, Eskenazi B, Warner M, Samuels S, Vercellini P, Gavoni N, et al. Uterine leiomyoma and menstrual cycle characteristics in a population-based cohort study. Hum Reprod. 2004;19(10):2350–5. doi: 10.1093/humrep/deh407 15242998

[23] Parazzini F, LaVecchia C, Negri E, Cecchetti G, Fedele L. Epidemiologic Characteristics of Women With Uterine Fibroids: A Case-Control Study. Vol. 72, Obstetrics & Gynaecology. 1988. p. 853–7. doi: 10.1097/00006250-198812000-00008 3186092

[24] Ross RK, Pike MC, Vessey MP, Bull D, Yeates D, Casagrande JT. Risk factors for uterine fibroids: reduced risk associated with oral contraceptives. Br Med J (Clin Res Ed) [Internet]. 1986;293(6543):359–62. Available from: http://www.pubmedcentral.nih.gov/articlerender.fcgi?artid=1341047&tool=pmcentrez&rendertype=abstract. doi: 10.1136/bmj.293.6543.359 3730804 PMC1341047

[25] Martin CL, Huber LRB, Thompson ME, Racine EF. Serum micronutrient concentrations and risk of uterine fibroids. J Womens Health (Larchmt) [Internet]. 2011 Jun [cited 2016 Sep 18];20(6):915–22. Available from: http://www.ncbi.nlm.nih.gov/pubmed/21671776. doi: 10.1089/jwh.2009.1782 21671776

[26] Faerstein E, Szklo M, Rosenshein N. Risk factors for uterine leiomyoma: a practice-based case-control study. Am J Epidemiol. 2001;153:1–10. 11159139 10.1093/aje/153.1.1

[27] Chen C-R. Risk Factors for Uterine Fibroids among Women Undergoing Tubal Sterilization. Am J Epidemiol [Internet]. 2001;153(1):20–6. Available from: http://aje.oxfordjournals.org/cgi/content/long/153/1/20. doi: 10.1093/aje/153.1.20 11159141

[28] Chiaffarino F, Parazzini F, La Vecchia C, Marsico S, Surace M, Ricci E. Use of oral contraceptives and uterine fibroids: results from a case-control study. Br J Obstet Gynaecol. 1999;106(August):857–60. doi: 10.1111/j.1471-0528.1999.tb08409.x 10453838

[29] Marshall LM, Spiegelman D, Goldman MB, Manson JE, Colditz GA, Barbieri RL, et al. A prospective study of reproductive factors and oral contraceptive use in relation to the risk of uterine leiomyomata. Fertil Steril. 1998;70(3):432–9. doi: 10.1016/s0015-0282(98)00208-8 9757871

[30] Samadi AR, Lee NC, Dana Flanders W, Boring JR, Parris EB. Risk factors for self-reported uterine fibroids: A case-control study. Am J Public Health. 1996;86(6):858–62. doi: 10.2105/ajph.86.6.858 8659663 PMC1380408

[31] Lumbiganon P, Rugpao S, Phandhu-fung S, Laopaiboon M, Vudhikamraksa N, Werawatakul Y. Protective effect of depot-medroxyprogesterone acetate on surgically treated uterine leiomyomas: A multicentre case-control study. Br J Obstet Gynaecol [Internet]. 1995 Sep [cited 2016 Sep 17];103(9):909–14. Available from: http://www.ncbi.nlm.nih.gov/pubmed/8813312. 10.1111/j.1471-0528.1996.tb09911.x 8813312

[32] Parazzini F, Negri E, Vecchia C, Fedele L, Rabaiotti M, Luchini L. Oral contraceptive use and risk of uterine fibroids. Obstet Gynaecol. 1992;(79):430–3. doi: 10.1097/00006250-199203000-00021 1738528

[33] Hoffman SR, Farland L V, Doll KM, Nicholson WK, Wright MA, Robinson WR, et al. The epidemiology of gynaecologic health: contemporary opportunities and challenges. J Epidemiol Community Heal. 2020;1–4. doi: 10.1136/jech-2019-213149 33109525 PMC8095335

[34] Marshall LM, Spiegelman D, Barbieri RL, Goldman MB, Manson JE, Colditz GA, et al. Variation in the incidence of uterine leiomyoma among premenopausal women by age and race. Obs Gynecol [Internet]. 1997;90(6):967–73. Available from: http://www.ncbi.nlm.nih.gov/pubmed/9397113%5Cnhttp://ac.els-cdn.com/S0029784497005346/1-s2.0-S0029784497005346-main.pdf?_tid=6ea0459c-5f93-11e3-be85-00000aab0f02&acdnat=1386457437_e02eb331111dbd56f3b5cacd61615d6b. doi: 10.1016/s0029-7844(97)00534-6 9397113

[35] Stewart EA, Nicholson WK, Bradley L, Borah BJ. The burden of uterine fibroids for African-American women: results of a national survey. J Womens Health (Larchmt) [Internet]. 2013 Oct [cited 2017 Feb 5];22(10):807–16. Available from: http://www.ncbi.nlm.nih.gov/pubmed/24033092. doi: 10.1089/jwh.2013.4334 24033092 PMC3787340

[36] Eltoukhi H, Modi M, Weston M, Armstrong A, Stewart E. The Health Disparities of Uterine Fibroids for African American Women: A Public Health Issue. AM J Obs Gynecol. 2014;210(3):194–9. 10.1016/j.ajog.2013.08.008 PMC3874080 23942040

[37] Wechter ME, Stewart EA, Myers ER, Kho RM, Wu JM. Leiomyoma-related hospitalization and surgery: prevalence and predicted growth based on population trends. Am J Obstet Gynecol [Internet]. 2011 Nov [cited 2017 Feb 19];205(5):492.e1-5. Available from: http://www.ncbi.nlm.nih.gov/pubmed/22035951. doi: 10.1016/j.ajog.2011.07.008 22035951 PMC3746963

[38] Hoffman SR, Smith JS, Funk MJ, Poole C, Harmon QE, Nicholson WK, et al. Combined oral contraceptive utilization and uterine fibroid incidence and prevalence in the Study of Environment, Lifestyle, and Fibroids (SELF) [Internet]. 2019 [cited 2023 Dec 9]. Available from: https://cdr.lib.unc.edu/concern/dissertations/028711697?locale=en.

[39] Harmon QE, Umbach DM, Baird DD. Use of Estrogen-Containing Contraception Is Associated With Increased Concentrations of 25-Hydroxy Vitamin D. 2016;(August):1–8. doi: 10.1210/jc.2016-1658 27490916 PMC5010573

[40] Jukic AMZ, Upson K, Harmon QE, Baird DD. Increasing serum 25-hydroxyvitamin D is associated with reduced odds of long menstrual cycles in a cross-sectional study of African American women. Fertil Steril [Internet]. 2016;06450. Available from: http://www.ncbi.nlm.nih.gov/pubmed/26997249. 10.1016/j.fertnstert.2016.03.004 PMC4930882 26997249

[41] Harmon QE, Baird DD. Use of depot medroxyprogesterone acetate and prevalent leiomyoma in young African American women. Hum Reprod [Internet]. 2015;30(6):1499–504. Available from: http://humrep.oxfordjournals.org/content/30/6/1499.abstract. doi: 10.1093/humrep/dev069 25820696 PMC4447888

[42] Stewart EA. Epidemiology, clinical manifestations, diagnosis, and natural history of uterine leiomyomas (fibroids) [Internet]. UpToDate. 2016 [cited 2016 Sep 17]. Available from: http://www.uptodate.com/contents/epidemiology-clinical-manifestations-diagnosis-and-natural-history-of-uterine-leiomyomas-fibroids?source=machineLearning&search=uterine+fibroid&selectedTitle=2~150&sectionRank=2&anchor=H11#H11.

[43] Moshesh M, Peddada SD, Cooper T, Baird D. Intraobserver Variability in Fibroid Size Measurements. J Ultrasound Med [Internet]. 2014;33(7):1217–24. Available from: http://doi.wiley.com/10.7863/ultra.33.7.1217. 24958408 10.7863/ultra.33.7.1217 PMC5452979

[44] Dueholm M, Lundorf E, Hansen ES, Ledertoug S, Olesen F. Accuracy of magnetic resonance imaging and transvaginal ultrasonography in the diagnosis, mapping, and measurement of uterine myomas. Am J Obstet Gynecol [Internet]. 2002 Mar [cited 2016 Sep 17];186(3):409–15. Available from: http://www.ncbi.nlm.nih.gov/pubmed/11904599. doi: 10.1067/mob.2002.121725 11904599

[45] Hoffman SR, Nicholson WK, Smith JS, Jonsson Funk M, Hudgens MG, Poole C, et al. Reasons for hormonal contraceptive use in a cohort of African-American women living in the Detroit area. Contraception [Internet]. 2020;102(5):346–8. Available from: doi: 10.1016/j.contraception.2020.07.093 32768397 PMC7606661

[46] Ali MS, Groenwold RHH, Belitser S V., Pestman WR, Hoes AW, Roes KCB, et al. Reporting of covariate selection and balance assessment in propensity score analysis is suboptimal: A systematic review. J Clin Epidemiol [Internet]. 2015;68(2):122–31. Available from: 10.1016/j.jclinepi.2014.08.011. 25433444

[47] Wasserstein RL, Lazar NA. The ASA Statement on p-Values: Context, Process, and Purpose. Am Stat [Internet]. 2016 Apr 2 [cited 2023 Dec 9];70(2):129–33. Available from: https://www.tandfonline.com/doi/abs/10.1080/00031305.2016.1154108.

[48] Bulun SE, Moravek MB, Yin P, Ono M, Coon V JS, Dyson MT, et al. Uterine Leiomyoma Stem Cells: Linking Progesterone to Growth. Semin Reprod Med [Internet]. 2015 Aug 6 [cited 2023 Dec 9];33(5):357–65. Available from: https://pubmed.ncbi.nlm.nih.gov/26251118/. doi: 10.1055/s-0035-1558451 26251118

[49] Ishikawa H, Ishi K, Ann Serna V, Kakazu R, Bulun SE, Kurita T. Progesterone is essential for maintenance and growth of uterine leiomyoma. Endocrinology [Internet]. 2010 Jun [cited 2023 Dec 9];151(6):2433–42. Available from: https://pubmed.ncbi.nlm.nih.gov/20375184/. doi: 10.1210/en.2009-1225 20375184 PMC2875812

[50] Qin H, Lin Z, Vásquez E, Luan X, Guo F, Xu L. Association between obesity and the risk of uterine fibroids: a systematic review and meta-analysis. J Epidemiol Community Health. 2021;75(2):197–204. doi: 10.1136/jech-2019-213364 33067250

[51] Hill AB. The Environment and Disease: Association or Causation? Proc ofthe R Soc ofMedicine [Internet]. 1965;58(5):295–300. Available from: https://www.ncbi.nlm.nih.gov/pmc/articles/PMC1898525/. 14283879 10.1177/003591576505800503 PMC1898525

[52] Westreich D, Cates J, Cohen M, Weber KM, Seidman D, Cropsey K, et al. Smoking, HIV, and risk of pregnancy loss (see “Population intervention estimates”). AIDS. 2017 Feb 20;31(4):553–60. 27902507 10.1097/QAD.0000000000001342 PMC5263172

[53] Westreich D. From Patients to Policy: Population Intervention Effects in Epidemiology. Epidemiology [Internet]. 2017 Jul 1 [cited 2021 May 13];28(4):525–8. Available from: /pmc/articles/PMC5453818/. doi: 10.1097/EDE.0000000000000648 28282339 PMC5453818

[54] Westreich D. From exposures to population interventions: Pregnancy and response to HIV therapy (see “Limitations of prevoius work”). Am J Epidemiol [Internet]. 2014 [cited 2021 May 13];179(7):797–806. Available from: /pmc/articles/PMC3969531/. 24573538 10.1093/aje/kwt328 PMC3969531

[55] Spangler L, Ichikawa LE, Hubbard RA, Operskalski B, LaCroix AZ, Ott SM, et al. A comparison of self-reported oral contraceptive use and automated pharmacy data in perimenopausal and early postmenopausal women. Ann Epidemiol. 2015. doi: 10.1016/j.annepidem.2014.10.009 25453353 PMC4300298

[56] Ray WA. Evaluating medication effects outside of clinical trials: new-user designs. Am J Epidemiol [Internet]. 2003 Nov 1 [cited 2018 Aug 6];158(9):915–20. Available from: http://www.ncbi.nlm.nih.gov/pubmed/14585769. doi: 10.1093/aje/kwg231 14585769

