# Supplementary material for: Combined oral contraceptive utilization and uterine fibroid incidence: A prospective study in a cohort of African-American women
Source: PLoS One. 2024 May 23;19(5):e0303823. doi: 10.1371/journal.pone.0303823 (PMC11115284; doi:10.1371/journal.pone.0303823)
Supplement: S1 File — (DOCX) [file pone.0303823.s002.docx]

**Supplemental Material**

- S1 Appendix – Propensity score-based methods
- S2 Table – Sample sizes and distribution of weights
- S3 Appendix – Covariate balance & propensity score curves
- S4 Appendix – P-value functions

**Appendix S1 – Propensity Score-based Methods**

**Variable selection for PS model**

Substantive knowledge and directed acyclic graphs (DAGs) were used to determine which covariates to place into the propensity score and censoring models. Two experienced fibroid epidemiologists (DBB & QEH) and one practicing OB/GYN (WKN) were consulted.

**Propensity score estimation**

Propensity scores were estimated by logistic regression and multinomial logistic regression for binary and non-binary exposures, respectively.^1,2^ The following variables were included in all propensity score models: age in years (continuous), age at menarche (<11 years), Depo-Provera duration of and years since last use (never use, short/past, long/past, short/recent, long/recent), total implant and H-IUD duration of use (>24 months), years since last birth (<5 years, 5-9.99 years, >10 years and no birth), parity (nulliparous or never pregnant, 1 birth, 2 births, ≥3 births), BMI (>30, remaining values according to tertile), and education (Bachelor’s degree or higher).

**Censoring weights estimation**

Propensity scores for remaining in the study were estimated by logistic regression. For each model, we included the exposure of interest, all covariates used to estimate the PS for the exposure of interest, and annual household income (<$20,000, $20,000-49,999, and >$50,000), baseline employment status (not employed, employed <30 hours per week, employed >30 hours per week), smoking history (never smoked, former smoker, current smoker of <10 /day, current smoker of >10/day) and history of heavy “gushing” type menstrual bleeding (yes/no).

**Applying the PS method: PS weighting**

Inverse probability (IP) weights were constructed for all exposures, and censoring. Standardized morbidity ratio (SMR) weights were constructed for COC use. For incidence analyses, the IP and SMR weights were multiplied by the inverse probability of censoring weights. The range (mean, max, min) of unstabilized and stabilized weights are available in **Appendix B**. Weights were not truncated. Covariate balance tables also appear in **Appendix C**.

**Balance assessment**

Absolute standardized differences (ASD) were used to assess covariate balance with an a priori threshold of 0.1. Propensity score distributions were examined before and after weighting to assess for positivity and covariate balance, respectively. Since our stabilized and unstabilized weights produced the same point estimates and confidence intervals, we report the covariate balance and findings for the unstabilized weights.

**Treatment effect estimation**

Weighted log-binomial regression models were used to estimate risk ratios for uterine fibroids. No additional covariates were included in the weighted models. Confidence intervals for weighted models were generated using robust variance (“sandwich”) estimator by use of the SAS REPEAT statement. Multivariable log-binomial regression models with the same covariates as used in the propensity score models were run for comparison.

**Interpretation of effect estimates**

The risk ratios computed for age at first COC use, duration of use, years since last use, and joint duration of and years since last use represent estimates of the average treatment effect (ATE), i.e., the weights balance the covariate distribution to reflect the distribution among all COC users across all levels of COC use. For each comparison made, the corresponding counterfactual comparison to our weighted analysis was, *“if the entire sample of COC users had used at this level versus the referent level.”* The risk ratios computed for ever-use of COCs represent estimates of the average effect in the treated (ATT), i.e., the weights balance the covariate distribution to reflect the distribution among COC users (i.e., the “treated” group). The relevant counterfactual comparison to our weighted analysis was, *“if all COC users had not used COCs.”*

**References**

1. Hoffman SR. Inverse probability weighting for non-binary exposures: simple example in Excel and SAS. 2019. https://github.com/srhoffma/non_binary_exposure_IPW.

2. Naimi AI, Moodie EEM, Auger N, Kaufman JS. Constructing inverse probability weights for continuous exposures: A comparison of methods. *Epidemiology*. 2014;25(2):292-299. doi:10.1097/EDE.0000000000000053

**Appendix S2 – Sample sizes and distribution of weights for all models**

| **Table B1.** Incidence Models | | | | | | | |
| --- | --- | --- | --- | --- | --- | --- | --- |
|  | **Age-adjusted** | **Fully adjusted** | |  |  |  |  |
| **Model** | **MVR** | **IPW/SMR**^†^ | **MVR** | **IPW** | **SIPW/SMRW** | **Censoring weight** | **Final weight**^†^ |
|  | **N** | **N** | **N** | **Mean [Range]** | **Mean [Range]** | **Mean [Range]** | **Mean [Range]** |
| Ever use | 1,110 | 1,098* | 1,109 | NA | 1.39 [0.91, 6.37]* | 1.00 [0.89, 1.33] | 1.39 [0.87, 6.81]* |
| Age at first use | 777 | 769 | 777 | 1.99 [1.18, 6.49] | 1.00 [0.66, 2.27] | 1.00 [0.87, 1.49] | 1.98 [1.11, 6.58] |
| Duration of use | 777 | 769 | 777 | 4.00 [1.65, 21.04] | 1.00 [0.44, 5.58] | 1.00 [0.88, 1.42] | 3.99 [1.51, 22.89] |
| Years since last use | 754 | 747 | 754 | 4.06 [1.09, 45.72] | 1.01 [0.27, 8.79] | 1.00 [0.89, 1.39] | 4.04 [1.03, 43.83] |
| Characteristics of use^‡^ | 754 | 747 | 754 | 3.95 [1.49, 21.34] | 0.99 [0.40, 5.75] | 1.00 [0.88, 1.40] | 3.93 [1.43, 21.23] |
| *SMR weights  ^†^Includes censoring weights. For Ever use, final weights consisted of SMR multiplied by the censoring weight. For all other exposures, final weight consisted of IPW multiplied by censoring weight.  ^‡^Joint duration of and years since last use. Duration of use was characterized as short (<2 years) or long (>2 years) and years since last use was characterized as recent (<5 years) or past (>5 years), based on available data, creating four joint categories.  **Abbreviations:** MVR, multivariable logistic regression; IPW, inverse probability weighting; SMR, standardized morbidity ratio; SIPW, stabilized inverse probability weight; SMRW, standardized morbidity ratio weight. | | | | | | | |

**Appendix S3 – Covariate Balance & Propensity Score Curves for Incidence Models**

**Exposure 1: Ever used COCs**

| **Table C1.** Covariate balance before and after SMR weighting for Ever/Never COC use among 1,308 SELF participants who were fibroid-free at enrollment | | | | | | | |
| --- | --- | --- | --- | --- | --- | --- | --- |
|  | **Unweighted** | | |  | **SMR & Censoring Weighted** | | |
| **Variable Name** | **Never COCs** | **Ever COCs** | **ASD** |  | **Never COCs** | **Ever COCs** | **ASD** |
| Age at enrollment | 27.57 | 28.73 | 0.34 |  | 28.65 | 28.75 | 0.02 |
| BMI category | 2.11 | 2.21 | 0.09 |  | 2.20 | 2.21 | 0.01 |
| Depo-Provera use | 1.41 | 1.28 | 0.08 |  | 1.30 | 1.26 | 0.02 |
| H-IUD/Implant use | 0.06 | 0.07 | 0.04 |  | 0.08 | 0.07 | 0.01 |
| Bachelor’s degree | 0.20 | 0.28 | 0.18 |  | 0.27 | 0.28 | 0.03 |
| Menarche age < 11 yrs. | 0.16 | 0.18 | 0.03 |  | 0.17 | 0.17 | 0.00 |
| Parity category | 1.13 | 1.22 | 0.08 |  | 1.25 | 1.21 | 0.03 |
| Years since last birth | 1.12 | 1.07 | 0.06 |  | 1.01 | 1.08 | 0.06 |
| **Abbreviations:** COCs, combined oral contraceptives; SMR, standardized morbidity ratio**;** SELF, study of environment lifestyle and fibroids; ASD, absolute standardized difference; BMI, body mass index; H-IUD, hormonal intrauterine device. | | | | | | | |

**Figure C1.** Unweighted & weighted PS distributions for Ever/Never Incidence Censor/SMR weighted model.

**Appendix S3 – Covariate Balance & Propensity Score Curves for Incidence Models (Cont’d)**

**Exposure 2: Age at first COC use**

| **Table C2.** Covariate balance before and after IP weighting for Age at first COC use among 913 COC users who were fibroid-free at enrollment in SELF | | | | | | | |
| --- | --- | --- | --- | --- | --- | --- | --- |
|  | **Unweighted** | | |  | **IP & Censoring Weighted** | | |
| **Variable Name** | **AFU < 17 years** | **AFU > 17 years** | **ASD** |  | **AFU < 17 years** | **AFU > 17 years** | **ASD** |
| Age at enrollment | 28.64 | 28.78 | 0.04 |  | 28.79 | 28.66 | 0.03 |
| BMI category | 2.35 | 2.12 | 0.21 |  | 2.21 | 2.19 | 0.01 |
| Depo-Provera use | 1.40 | 1.22 | 0.11 |  | 1.30 | 1.26 | 0.01 |
| H-IUD/Implant use | 0.07 | 0.08 | 0.05 |  | 0.08 | 0.08 | 0.00 |
| Bachelor’s degree | 0.21 | 0.32 | 0.26 |  | 0.28 | 0.28 | 0.01 |
| Menarche age < 11 yrs. | 0.18 | 0.17 | 0.02 |  | 0.17 | 0.17 | - |
| Parity category | 1.32 | 1.17 | 0.13 |  | 1.23 | 1.19 | 0.02 |
| Years since last birth | 1.05 | 1.08 | 0.04 |  | 1.06 | 1.07 | 0.01 |
| **Abbreviations:** COCs, combined oral contraceptives; IP, inverse probability**;** SELF, study of environment lifestyle and fibroids; AFU, age at first use; ASD, absolute standardized difference; BMI, body mass index; H-IUD, hormonal intrauterine device. | | | | | | | |

**Figure C2.** Unweighted & weighted PS distributions for age at first COC use incidence Censoring/IP weighted model.

**Appendix S3 – Covariate Balance & Propensity Score Curves for Incidence Models (Cont’d)**

**Exposure 3: Duration of COC use**

| **Table C3a.** Unweighted covariate balance table for Duration of COC use among 913 COC users who were fibroid-free at enrollment in SELF | | | | | | | |
| --- | --- | --- | --- | --- | --- | --- | --- |
| **Variable Name** | **< 1 year (0)** | **1 - 1.99 years (1)** | **2 - 4.99 years (2)** | **5 + years (3)** | **ASD**  **1 vs. 0** | **ASD**  **2 vs. 0** | **ASD**  **3 vs. 0** |
| Age at enrollment | 28.27 | 28.63 | 28.67 | 29.38 | 0.10 | 0.12 | 0.33 |
| BMI category | 2.19 | 2.14 | 2.23 | 2.24 | 0.04 | 0.04 | 0.05 |
| Depo-Provera use | 1.56 | 1.37 | 1.36 | 0.83 | 0.12 | 0.12 | 0.48 |
| H-IUD/Implant use | 0.09 | 0.09 | 0.05 | 0.06 | 0.01 | 0.16 | 0.12 |
| Bachelor’s degree | 0.17 | 0.26 | 0.28 | 0.43 | 0.24 | 0.28 | 0.59 |
| Menarche age < 11 yrs. | 0.19 | 0.18 | 0.16 | 0.17 | 0.04 | 0.09 | 0.06 |
| Parity category | 1.43 | 1.20 | 1.25 | 0.96 | 0.20 | 0.16 | 0.44 |
| Years since last birth | 0.92 | 1.10 | 1.11 | 1.17 | 0.21 | 0.23 | 0.29 |

| **Table C3b.** IP and censoring weighted covariate balance table for Duration of COC use among 913 COC users who were fibroid-free at enrollment in SELF | | | | | | | |
| --- | --- | --- | --- | --- | --- | --- | --- |
| **Variable Name** | **< 1 year (0)** | **1 - 1.99 years (1)** | **2 - 4.99 years (2)** | **5 + years (3)** | **ASD**  **1 vs. 0** | **ASD**  **2 vs. 0** | **ASD**  **3 vs. 0** |
| Age at enrollment | 28.67 | 28.67 | 28.67 | 28.76 | 0.00 | 0.00 | 0.01 |
| BMI category | 2.22 | 2.19 | 2.21 | 2.21 | 0.02 | 0.01 | 0.01 |
| Depo-Provera use | 1.25 | 1.31 | 1.31 | 1.37 | 0.02 | 0.02 | 0.04 |
| H-IUD/Implant use | 0.08 | 0.08 | 0.07 | 0.10 | 0.00 | 0.03 | 0.04 |
| Bachelor’s degree | 0.27 | 0.28 | 0.28 | 0.26 | 0.01 | 0.01 | 0.01 |
| Menarche age < 11 yrs. | 0.17 | 0.18 | 0.18 | 0.19 | 0.01 | 0.01 | 0.03 |
| Parity category | 1.17 | 1.26 | 1.20 | 1.27 | 0.04 | 0.01 | 0.05 |
| Years since last birth | 1.12 | 1.05 | 1.06 | 1.02 | 0.04 | 0.04 | 0.06 |
| **Abbreviations:** COCs, combined oral contraceptives; IP, inverse probability**;** SELF, study of environment lifestyle and fibroids; ASD, absolute standardized difference; BMI, body mass index; H-IUD, hormonal intrauterine device. | | | | | | | |

**Figure C3.** Unweighted & weighted PS distributions for duration of COC use incidence Censoring/IP weighted model.

**Appendix S3 – Covariate Balance & Propensity Score Curves for Incidence Models (Cont’d)**

**Exposure 4: Years since last COC use**

**Table C4a.** Unweighted covariate balance for years since last COC use among 913 COC users who were fibroid-free at enrollment in SELF

| **Variable Name** | **Current users (0)** | **1-2 years (1)** | **3-4 years (2)** | **5+ years (3)** | **ASD**  **1 vs. 0** | **ASD**  **2 vs. 0** | **ASD**  **3 vs. 0** |
| --- | --- | --- | --- | --- | --- | --- | --- |
| Age at enrollment | 27.88 | 27.56 | 27.64 | 29.77 | 0.10 | 0.07 | 0.56 |
| BMI category | 2.12 | 2.20 | 2.24 | 2.24 | 0.07 | 0.11 | 0.11 |
| Depo-Provera use | 0.81 | 1.50 | 0.99 | 1.50 | 0.46 | 0.13 | 0.45 |
| H-IUD/Implant use | 0.05 | 0.04 | 0.13 | 0.09 | 0.04 | 0.29 | 0.18 |
| Bachelor’s degree | 0.38 | 0.32 | 0.29 | 0.20 | 0.11 | 0.17 | 0.38 |
| Menarche age < 11 y | 0.14 | 0.16 | 0.19 | 0.19 | 0.06 | 0.12 | 0.13 |
| Parity category | 0.88 | 0.99 | 0.84 | 1.53 | 0.11 | 0.04 | 0.62 |
| Years since last birth | 1.17 | 1.12 | 1.16 | 0.99 | 0.06 | 0.01 | 0.20 |

**Table C4b.** Censoring Weighted and IPW Weighted covariate balance for years since last COC use among 913 COC users who were fibroid-free at enrollment in SELF

| **Variable Name** | **Current users (0)** | **1-2 years (1)** | **3-4 years (2)** | **5+ years (3)** | **ASD**  **1 vs. 0** | **ASD**  **2 vs. 0** | **ASD**  **3 vs. 0** |
| --- | --- | --- | --- | --- | --- | --- | --- |
| Age at enrollment | 29.09 | 28.51 | 29.23 | 28.89 | 0.07 | 0.01 | 0.03 |
| BMI category | 2.32 | 2.20 | 2.20 | 2.23 | 0.05 | 0.04 | 0.04 |
| Depo-Provera use | 1.49 | 1.33 | 1.25 | 1.29 | 0.04 | 0.06 | 0.07 |
| H-IUD/Implant use | 0.08 | 0.10 | 0.07 | 0.08 | 0.02 | 0.03 | 0.01 |
| Bachelor’s degree | 0.26 | 0.27 | 0.30 | 0.26 | 0.01 | 0.04 | 0.00 |
| Menarche age < 11 y | 0.16 | 0.15 | 0.20 | 0.18 | 0.01 | 0.03 | 0.02 |
| Parity category | 1.27 | 1.03 | 1.30 | 1.24 | 0.10* | 0.01 | 0.01 |
| Years since last birth | 1.14 | 1.16 | 1.02 | 1.07 | 0.01 | 0.05 | 0.05 |
| *0.09992 | | | | | | | |

**Abbreviations:** COCs, combined oral contraceptives; IP, inverse probability**;** SELF, study of environment lifestyle and fibroids; ASD, absolute standardized difference; BMI, body mass index; H-IUD, hormonal intrauterine device.

**Figure C4.** Unweighted & weighted PS distributions for years since last COC use incidence Censoring/IP weighted model.

**Appendix S3 – Covariate Balance & Propensity Score Curves for Incidence Models (Cont’d)**

**Exposure 5: Joint duration of and years since last COC use**

**Table C5a.** Unweighted covariate balance for joint duration of and years since last COC use among 913 COC users who were fibroid-free at enrollment in SELF

| **Variable Name** | **Short/Past (0)** | **Short/Recent (1)** | **Long/Past (2)** | **Long/Recent (3)** | **ASD**  **1 vs. 0** | **ASD**  **2 vs. 0** | **ASD**  **3 vs. 0** |
| --- | --- | --- | --- | --- | --- | --- | --- |
| Age at enrollment | 29.45 | 26.88 | 30.21 | 28.27 | 0.78 | 0.24 | 0.35 |
| BMI category | 2.20 | 2.14 | 2.30 | 2.19 | 0.05 | 0.10 | 0.00 |
| Depo-Provera use | 1.61 | 1.28 | 1.34 | 0.91 | 0.20 | 0.17 | 0.45 |
| H-IUD/Implant use | 0.11 | 0.08 | 0.07 | 0.05 | 0.08 | 0.12 | 0.22 |
| Bachelor’s degree | 0.15 | 0.27 | 0.28 | 0.39 | 0.28 | 0.30 | 0.55 |
| Menarche age < 11 y | 0.20 | 0.17 | 0.18 | 0.15 | 0.05 | 0.03 | 0.12 |
| Parity category | 1.60 | 0.96 | 1.43 | 0.87 | 0.59 | 0.15 | 0.68 |
| Years since last birth | 0.93 | 1.07 | 1.08 | 1.21 | 0.16 | 0.18 | 0.32 |

**Table C5b.** Censoring Weighted and IPW Weighted covariate balance for joint duration of and years since last COC use among 913 COC users who were fibroid-free at enrollment in SELF

| **Variable Name** | **Short/Past (0)** | **Short/Recent (1)** | **Long/Past (2)** | **Long/Recent (3)** | **ASD**  **1 vs. 0** | **ASD**  **2 vs. 0** | **ASD**  **3 vs. 0** |
| --- | --- | --- | --- | --- | --- | --- | --- |
| Age at enrollment | 28.90 | 28.58 | 28.83 | 29.03 | 0.04 | 0.01 | 0.02 |
| BMI category | 2.21 | 2.20 | 2.29 | 2.23 | 0.01 | 0.04 | 0.01 |
| Depo-Provera use | 1.25 | 1.32 | 1.39 | 1.38 | 0.02 | 0.04 | 0.04 |
| H-IUD/Implant use | 0.08 | 0.09 | 0.07 | 0.08 | 0.01 | 0.02 | 0.01 |
| Bachelor’s degree | 0.26 | 0.30 | 0.26 | 0.27 | 0.04 | 0.01 | 0.01 |
| Menarche age < 11 y | 0.18 | 0.16 | 0.18 | 0.19 | 0.03 | 0.00 | 0.01 |
| Parity category | 1.21 | 1.11 | 1.24 | 1.19 | 0.04 | 0.02 | 0.01 |
| Years since last birth | 1.11 | 1.15 | 1.01 | 1.13 | 0.03 | 0.05 | 0.01 |

**Abbreviations:** COCs, combined oral contraceptives; IP, inverse probability**;** SELF, study of environment lifestyle and fibroids; ASD, absolute standardized difference; BMI, body mass index; H-IUD, hormonal intrauterine device.

**Figure C5.** Unweighted & weighted PS distributions for JOINT duration of and years since last COC use incidence Censoring/IP weighted model.

**Appendix S4 – P-value functions**

Two-sided p-value functions for the weighted risk ratios (wRR) from the IPTW/SMR weighted comparisons:
